# Design and Development of Diabetes Management System Using Machine Learning

**DOI:** 10.1155/2020/8870141

**Published:** 2020-07-16

**Authors:** Robert A. Sowah, Adelaide A. Bampoe-Addo, Stephen K. Armoo, Firibu K. Saalia, Francis Gatsi, Baffour Sarkodie-Mensah

**Affiliations:** ^1^Department of Computer Engineering, University of Ghana, P.O. Box LG 77, Legon, Accra-, Ghana; ^2^Department of Food Process Engineering, And Department of Nutrition and Food Science, University of Ghana, P.O. Box LG 77, Legon, Accra-, Ghana; ^3^Department of Engineering and Computer Science, Ashesi University, Berekuso, Eastern Region, Ghana

## Abstract

This paper describes the design and implementation of a software system to improve the management of diabetes using a machine learning approach and to demonstrate and evaluate its effectiveness in controlling diabetes. The proposed approach for this management system handles the various factors that affect the health of people with diabetes by combining multiple artificial intelligence algorithms. The proposed framework factors the diabetes management problem into subgoals: building a Tensorflow neural network model for food classification; thus, it allows users to upload an image to determine if a meal is recommended for consumption; implementing K-Nearest Neighbour (KNN) algorithm to recommend meals; using cognitive sciences to build a diabetes question and answer chatbot; tracking user activity, user geolocation, and generating pdfs of logged blood sugar readings. The food recognition model was evaluated with cross-entropy metrics that support validation using Neural networks with a backpropagation algorithm. The model learned features of the images fed from local Ghanaian dishes with specific nutritional value and essence in managing diabetics and provided accurate image classification with given labels and corresponding accuracy. The model achieved specified goals by predicting with high accuracy, labels of new images. The food recognition and classification model achieved over 95% accuracy levels for specific calorie intakes. The performance of the meal recommender model and question and answer chatbot was tested with a designed cross-platform user-friendly interface using Cordova and Ionic Frameworks for software development for both mobile and web applications. The system recommended meals to meet the calorific needs of users successfully using KNN (with *k* = 5) and answered questions asked in a human-like way. The implemented system would solve the problem of managing activity, dieting recommendations, and medication notification of diabetics.

## 1. Introduction

Diabetes is a chronic disease that occurs either when the pancreas does not produce enough insulin or when the body cannot effectively use the insulin it produces. Insulin is a hormone that regulates blood sugar. Hyperglycaemia, or raised blood sugar, is a common effect of uncontrolled diabetes and, over time, leads to severe damage to many of the body's systems, especially the nerves and blood vessels [1]. In 2014, 8.5% of adults aged 18 years and older had diabetes worldwide. In 2016, diabetes was the direct cause of 1.6 million deaths, and in 2012 high blood glucose was the cause of another 2.2 million deaths [1–4]. There are two types of diabetes, namely, Type 1 and Type 2. Type 1 diabetes (previously known as insulin-dependent, juvenile, or childhood-onset) is characterized by deficient insulin production and requires daily administration of insulin. The cause of Type 1 diabetes is not known, and it is not preventable with current knowledge [1] [5]. Type 2 diabetes (formerly called noninsulin-dependent, or adult-onset) results from the body's ineffective use of insulin. Type 2 diabetes comprises what the majority of people with diabetes around the world have and is mostly the result of excess body weight and physical inactivity [1, 5–8]. According to the World Health Organization (WHO), to help prevent type 2 diabetes and its complications, people should achieve and maintain healthy body weight; be physically active—at least 30 minutes of regular, moderate-intensity activity on most days. More activity is required for weight control, eat a healthy diet, avoiding sugar and saturated fats intake, and avoid tobacco use—smoking increases the risk of diabetes and cardiovascular diseases [1, 8, 9]. Treatment of diabetes involves diet and physical activity, along with lowering blood glucose and the levels of other known risk factors that damage blood vessels [8–12].

It is evident from the WHO reviewed literature that the incidence of diabetes mellitus is ever increasing throughout the world in both developed and developing countries. A significant number of people living in both developed and developing countries are ever becoming sedentary. Moreover, although there is evidence that the complications of diabetes can be prevented, there are still diabetics who lack the required knowledge and skills to manage and control their condition using available technology for healthy living and changed lifestyles.

Changing lifestyles require deliberate effort. Therefore, diabetics must take the ultimate responsibility for their care and treatment using available technology-related systems. Technologies such as meal recommendation systems, physical activity monitoring and tracking, notification systems for taking drugs, and interactive chatbots for answering questions that they may have about their condition.

The advances in artificial intelligence (AI) and in particular machine learning and computer vision have made producing applications to automate tasks requiring intelligent behaviour, learning, and adaptation possible, hence, providing solutions to real-life problems such as diabetes management [11, 13–18]. AI systems can be applied in control, planning, and scheduling, answering diagnostic and consumer questions, handwriting recognition, speech, and facial recognition. They are now in routine use in various fields, including medicine, military, and engineering [19]. This paper reports the design and development of an artificial intelligence software system that incorporates the factors that are considered when managing diabetes. Emphasis is placed on the development of a meal recommendation system, activity tracking and monitoring, diabetes education through chatbots, as well as medication reminders. These tasks in diabetes management are developed into software modules to assist in personalized health assistant for diabetics. Also, central to the research work is the development of an educational platform that will provide answers to common questions of people living with diabetes. It will also allow doctors to gather data about patient behaviour and symptoms and intervene when patients are not following their regimens or have a flare-up in their disease condition. The diabetes management system aims at determining the required nutrition of patients and recommend meals to meet these needs, notify patients to take their medication on time, identify the factors that influence blood sugar, and educate patients on them. The developed system also encourages patients to exercise and keep track of their activities and keep doctors updated on the patient's condition by sending them pdfs of patient's data securely. These proposed objectives of the software modules help in delivering the required assistance in diabetes management through software engineering technology and machine learning.

The unique contributions of this paper are (1) design and development of a machine learning model for food recommendation system for diabetics using K-Nearest Neighbour (KNN) algorithm, (2) scheduling and reminding diabetic patients to take their medication and blood glucose readings for doctor's intervention via mobile app, (3) encouraging and tracking the activity of diabetic patients, and (4) providing an interactive visual interface to help them make meaning of their readings and establishing a sufficient connection between the doctor and the diabetic patient using e-mail and chatbots.  Section 2 presents the literature review on diabetes management and control using different techniques such as the artificial pancreas, machine learning food recommendation for blood glucose control, and management. It addresses the various machine learning techniques with implemented web technologies for diabetes management and control. It identifies the strengths and weaknesses of such systems. It proposes a novel method using the KNN machine learning algorithm as the core for food recommendation model with its associated modules for food recognition from images and calculation of calorie intake, development of chatbots using natural language processing for answering questions on diabetes, and offering general education on diabetes management, which is crucial.  Section 3 provides the novelty of the system design architecture and methodology, including the concepts, algorithms, and flowchart. It highlights the various modules with their design and procedures for implementation.  Section 4 focuses on the actual design implementations with experimental verification and corresponding integration of the developed modules. This section provides sufficient details on the testing and performance evaluation of the proposed food recommendation and recognition systems with its associated modules for efficient monitoring and control of diabetes. Finally, Section 5 provides conclusions on the system's design and methodology and relevant recommendations for future enhancements.

## 2. Literature Review

In references [2, 6], the authors developed a meal recommender system using the knowledge-based approach where the recommended meal will meet the users' needs based on knowledge of the required nutrients obtained from the user's details. They implemented their system using Genetic algorithm where a random number of meals were selected, and a fitness function calculated using the difference between calories in meals and calories required by the user. The researchers maintained that their genetic algorithm was an excellent choice because it found the optimum solution for scheduling diet for diabetics. But their algorithm failed to consider the amount of carbohydrates in the recommended meal and used just the calories to calculate the fitness score. This approach is not an ideal recommender for people with diabetes because carbohydrates in food affect blood sugar levels and must be included when recommending meals. They admitted that their system had a probabilistic weakness that resulted from the random selection of meals for their algorithm.

Mertz [3] reported an innovative technology developed for commercialization that combines four essential parts, namely, (1) a company-developed algorithm that incorporates artificial intelligence (AI) and machine learning, (2) a simple-to-use smartphone app, (3) a groundbreaking no-finger-stick glucose-monitoring technology, called FreeStyle Libre, and (4) a Bluetooth-enabled insulin pump. This development combines several technologies but lacks a meal recommender system.

Fico et al. [5] proposed a system that constructs three ontologies for exercise, meals, and user's data and integrates them to recommend meals, exercise, and send reminders to users. The integrated ontology imports the other domains and defines the relationship between them. This system offers practical treatment advice for diabetes patients and allows timely management of their blood sugar level, but lacks effective meal recommendation and food recognition capabilities. It does not provide activity tracking and interactive visual interface with a chatbot.

Researchers in [9] presented the SMARTDIAB platform for diabetes management. It combines the state-of-the-art methods in database (DB) technologies, communications, simulation algorithms, and data mining for managing Type 1 diabetes. It incorporated both hardware and software components for the efficient management of diabetes but lacks an effective meal recommender and food recognition capabilities.

Alian et al. [15] presented a personalized recommendation system to support diabetes self-management for American Indians by integrating users' ontological profile with general clinical diabetes recommendations and guidelines but lacks the development of a mobile application effective meal recommendation and food recognition capabilities. It does not provide activity tracking of the diabetic patient and does not have an interactive visual interface.

Alotaibi et al. [19] developed a diabetes management system using PHP and MySQL for the web application, Objective C for iPhone, and Java for Android. The system included a reminder module that used SMS to alert patients to take readings, an interface to log readings, and an artificial intelligence unit that determines the health level using Fuzzy logic. While this above system provides an excellent platform to keep doctors updated on patient condition, it does not include a module for diet and activity tracking. That is a limitation because diet and activity monitoring is very crucial in managing diabetes.

Zheng et al. [20] conducted a research study aimed at assessing the effectiveness of a simple outpatient diabetes self-management education programme. In the study, 60 patients with type 2 diabetes mellitus were randomly and equally allocated to the control group and the intervention group. There was no meal recommender system incorporated in this study.

The Chronic Care Model, presented in [21], incorporates core elements of team-centered care in chronic disease management. The study lacks activity tracking of the diabetic patient and an interactive visual interface, and it has no meal recommendation and food recognition capabilities available on a smartphone.

Phanich et al. [22] used clustering algorithms and self-organizing maps to group foods based on their nutritional content. They categorized foods into limited, normal, and avoidable foods. Their recommender system does not recommend meals; instead, it suggests healthy alternatives to replace food items in avoidable food categories. Although the system they implemented helps people with diabetes to avoid eating meals that are not good for their health, it does not take into account the details of the user. This can be a disadvantage because different people have different body types and activity levels in different Body Mass Index (BMI). Recommending the same meal for different users will have different implications on their blood sugar levels. Because the number of calories is not considered, weight management is not implemented in this system, and this may cause severe complications for a person with diabetes.

Yang et al. [23] proposed *Yum-me*, a personalized nutrient-based meal recommender system designed to meet individuals' nutritional expectations, dietary restrictions, and fine-grained food preferences. *Yum-me* provided a novel online learning framework that learns food preference from itemwise and pairwise image comparisons. Yum-me improves the recommendation acceptance rate by 42.63% but lacks the Diabetes question and answer chatbot to provide education on diabetes.

Toledo et al. [24] proposed a general framework for daily meal plan recommendations, incorporating simultaneous management of nutritional-aware and preference-aware information. It includes a prefiltering stage that uses AHPSort as a multicriteria decision analysis tool for filtering out foods that are not appropriate to user characteristics. While this development provides excellent meal recommendations based on simultaneous user nutritional-aware and preference-aware information, it lacks Diabetes questions and answers chatbot to provide education on diabetes as well as medicine reminder notification on smartphones.

Gu et al. [25] presented SugarMate, a smartphone-based blood glucose inference system, as a temporary alternative to continuous blood glucose monitors (CGM). In addition to the records of food, drug, and insulin intake, it leverages smartphone sensors to measure physical activities and sleep quality automatically. Even though evaluations on 112 users demonstrate that SugarMate implemented algorithm Md3RNN yields an average accuracy of 82.14%, significantly outperforming previous learning methods, it lacks the Diabetes question and answer chatbot to provide education on diabetes.

Mokdara et al. [26] presented an integration of a deep neural network with a recommendation system with Thai foods as a test domain. The proposed model extracts interested ingredients from the set of recipes of the user's favorite dishes but lacks the Diabetes questions and answers chatbot to provide education on diabetes as well as medicine reminder notification on smartphones.

Bianchini et al. [27] proposed the PREFer food recommender system, apt to provide users with personalized and healthy menus, taking into account both user's short/long-term preferences and medical prescriptions. Prescriptions classify the *ideal user*'*s nutrition behaviour* from the health point of view, with constraints imposed by the specific user's phenotype but lack Diabetes questions and answers chatbot to provide education on diabetes as well as medicine reminder notification on smartphones and food recognition capabilities.

Xie et al. [28] developed a mobile-based diabetes question-answering (Q&A) and early warning system named Dia-AID, assisting diabetes patients and populations at high risk. The Dia-AID system consists of three modules: large-scale multilanguage diabetes frequently asked question repository, a multimode fusion Q&A framework, and a health data management module. This system lacks the medicine reminder notification on smartphones, meal recommendations, and food recognition capabilities.

The analysis of these groups of works leads to the identification of several associated shortcomings. First, some of the reviewed literature did not focus on the processing of the users' meal preferences, diabetic education technology, medicine reminder notification, which are crucial elements in any diabetes management scenario. Furthermore, even though most of them considered food recommender systems for diabetes, food recognition capabilities based on computer vision with its associated rendering are not offered. Also, the incorporation of nutritional concepts and principles in the implemented computational models is not adequate. The existing diabetes management applications provided relevant general information search and management while ignoring counseling services offered by medical practitioners to diabetic patients via mobile apps with data logging capability, which were crucial for managing the health condition of diabetic patients.

## 3. System Design and Development

### 3.1. The System's Requirements and Design Analysis

In the design and development of the architecture for the diabetes management system, the clinical requirements and design analysis of the system were based on discussions with collaborators from the Department of Nutrition and Food Science of the University of Ghana and Kwame Nkrumah University of Science and Technology (KNUST). From these discussions, the diet type of patients was determined to be an essential approach suitable for the diabetes management system. The following functionalities were mentioned: (1) Scheduling and reminding diabetic patients to take their medication and blood glucose readings, (2) recommending healthy meals for diabetics to keep their blood glucose levels in check, (3) encouraging and tracking the activity of diabetic patients, (4) providing a visual interface to help them make meaning of their readings and establishing a sufficient connection between the doctor and the diabetic patient using e-mail.

Providing the diabetic patient with a data visualization tool to display the data in tables, charts, and an educational program for newly diagnosed and ongoing diabetes treatment is valuable for the treatment and management of diabetes.

### 3.2. System Architecture

**Figure 1:**
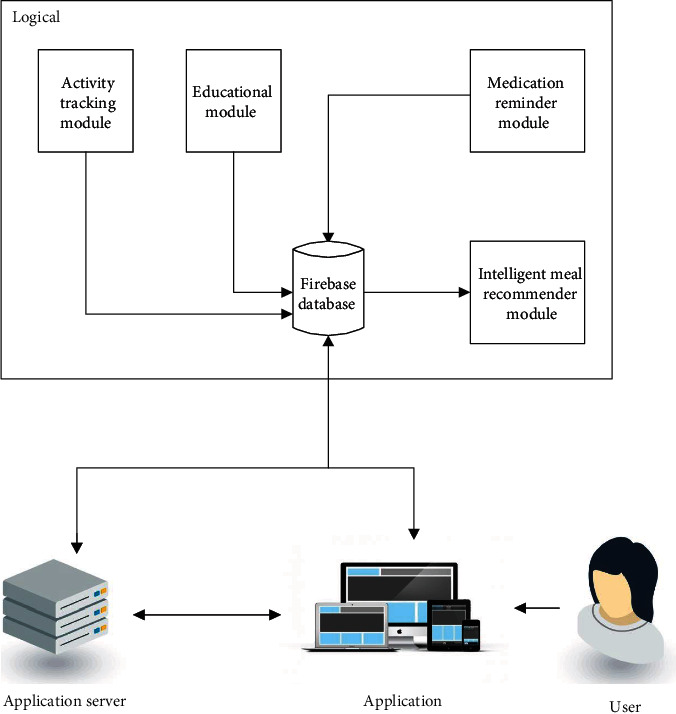
System architecture for the implemented system with all submodules.

The system architecture for the Diabetes Management System presented below in Figure 1 is the conceptual model that defines the structure, behavioural interactions, and multiple system views that underpins the system development. It presents the formal descriptions of the systems captured graphically that supports reasoning, and the submodules developed as well as the dataflows between the developed modules.

**Figure 2:**
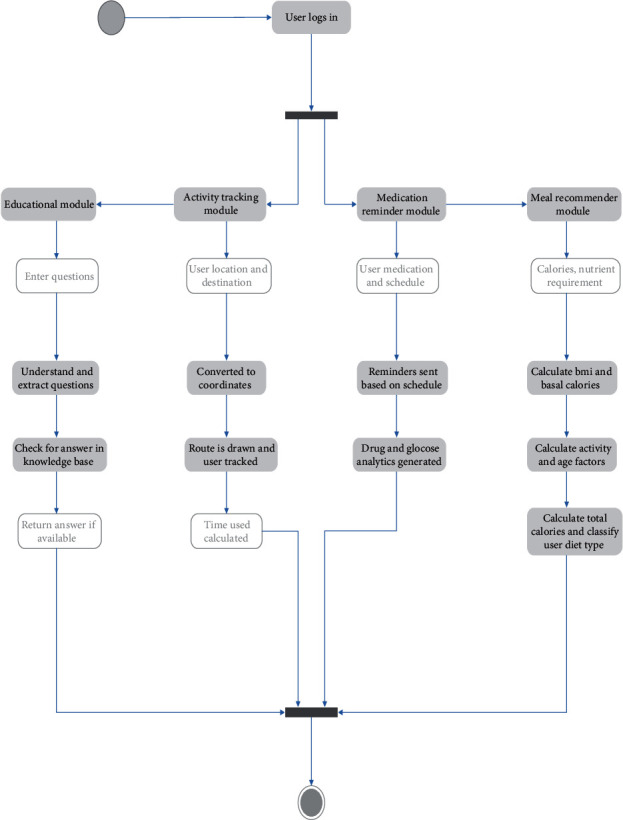
The activity flow diagram for the implemented system architecture.

Figure 1 presents the components of the system architecture, which comprises (1) Diabetes Intelligent Meal Recommender Module, (2) Educational Module for Diabetics with Food Recognition Engine, (3) Activity Tracking Module, and (4) Medication Reminder Module.  Figure 2 shows the corresponding activity flow diagram for the submodules developed.

### 3.3. The Diabetes Intelligent Meal Recommender Module

**Figure 3:**
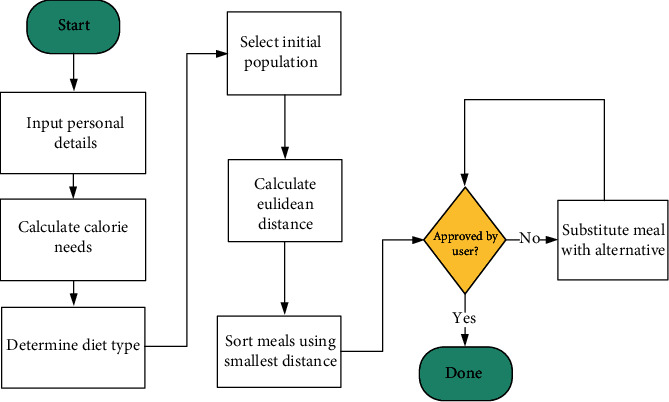
Flow chart for meal recommender system.

This module schedules a diet for diabetes management by generating whole meals for breakfast, lunch, and supper to meet the nutritional requirements of the diabetic patient. The recommender system uses the knowledge-based approach, where meals are recommended using the user's profile and a knowledge base. The steps followed for the meal recommendation system are outlined in the flowchart given below in Figure 3.

#### 3.3.1. Data Collection

The used data in this research consists of two data types, the patient data obtained from an interface provided to the user to input personal details like age, sex, weight, height, and level of activity. The food nutrition data was obtained from the Department of Nutrition and Food Science, University of Ghana, and from the MyFitnessPal database [29]. The diet type of the patient is determined from the obtained data, and calorie needs calculated using the Harris-Benedict's equation [30–33].

#### 3.3.2. Calorie Requirements Determination

**Figure 4:**
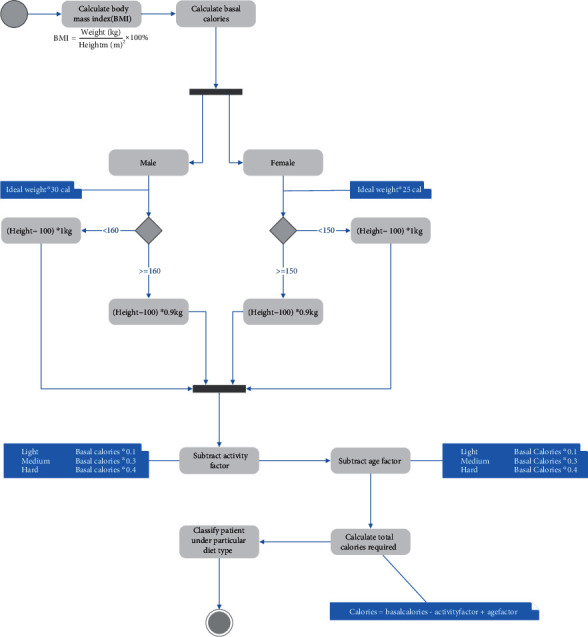
Flow chart for calorie requirements determination using the Harris Benedict's equation.

The steps followed using the Harris Benedict's equation are summarised below in the flowchart in Figure 4. The flowchart diagram encapsulates the actions taken for the Calorie requirements computation and determination.

#### 3.3.3. K-Nearest Neighbour Algorithm

**Figure 5:**
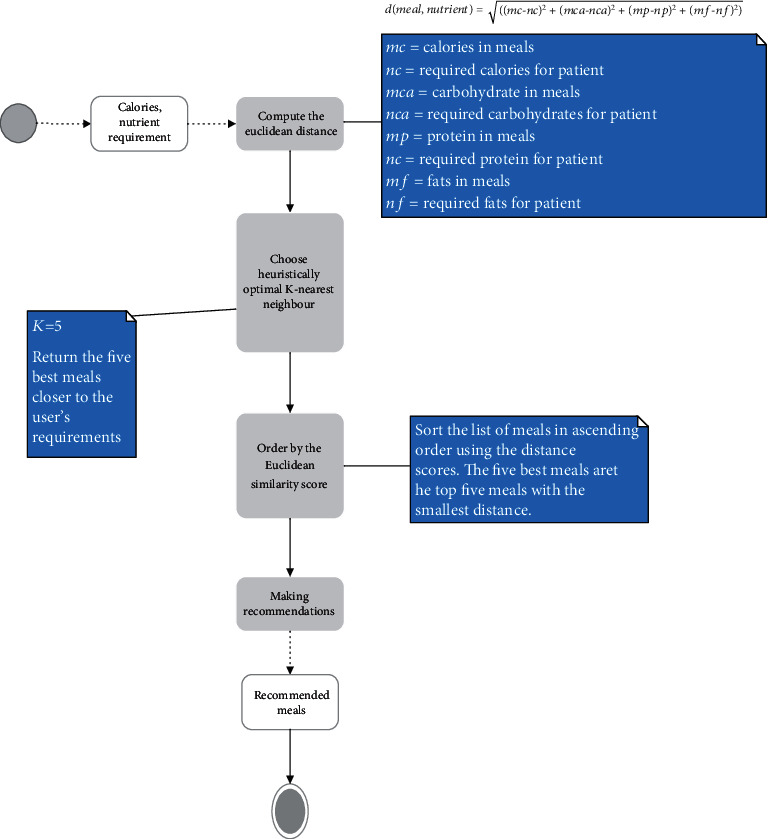
Flowchart for food recommendation using KNN.

K-Nearest Neighbour (KNN) is a supervised machine learning algorithm that stores available cases and predicts numerical targets based on a similarity measure. The KNN algorithm was used to implement KNN regression using a weighted average of the K nearest neighbours, weighted by the inverse of their distance. The actions taken are depicted in the activity flow diagram captured in the flowchart given below in Figure 5. Figure 5 presents the food recommendation engine using KNN.

### 3.4. The Educational Module with Food Recognition Capabilities

#### 3.4.1. Intelligent Question and Answer Bot

**Figure 6:**
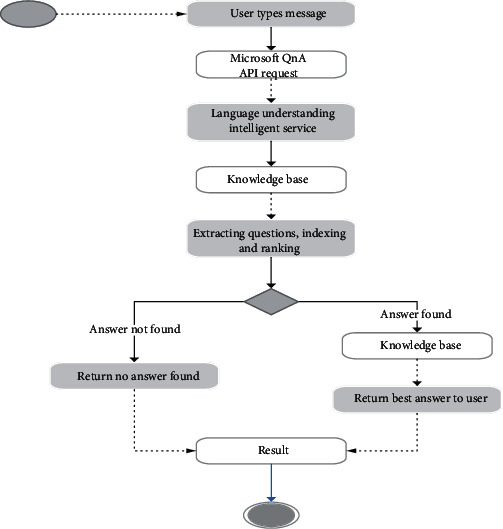
Flowchart for the implemented QnA chatbot.

Diabetes education is an essential part of diabetes management. This module has two units; the food recognition model and the intelligent question and answer chatbot. The Question-and-Answer chatbot calls the Microsoft QnA maker and language understanding intelligent service API [34], which uses natural language processing techniques to answer questions in the knowledge base, as shown in Figure 6 below. The knowledge base was trained with frequently asked questions from diabetes communities online and data collected from a diabetes specialist [1, 29]. The intelligent question and answer bot answer questions that patients have by simulating how a human would behave as a conversational partner. The key factor of this unit is to empower the patient with greater disease control and incorporate efficient self-management in his or her daily life using a chatbot. The flowchart for the QnA chatbot is given below in Figure 6.

First, information extraction and indexing are done before the operation of the application offline. When the application starts, the user is prompted to type his/her questions, the Rest API then determines how the API behaves like and uses HTTP requests to get, put, post, and delete data from the server. The Rest API follows a set of rules that interfaces the language understanding service running on the server. The language understanding application queries the knowledge base and extracts, searches, and ranks the user typed questions. If the typed question answer is found in the knowledge base, it ranks and indexes the answers and then returns the best answer to the user based on the pattern mining similarity garnered from training from the domain. For the question and answer bot, a Microsoft QnA service was created alongside a knowledge base which extracts question and answer pairs from diabetics pdfs and URLs of diabetes online forums. The model was trained and fine-tuned to improve performance and an API key generated to be called from the front end.

#### 3.4.2. Food Recognition Model

**Figure 7:**
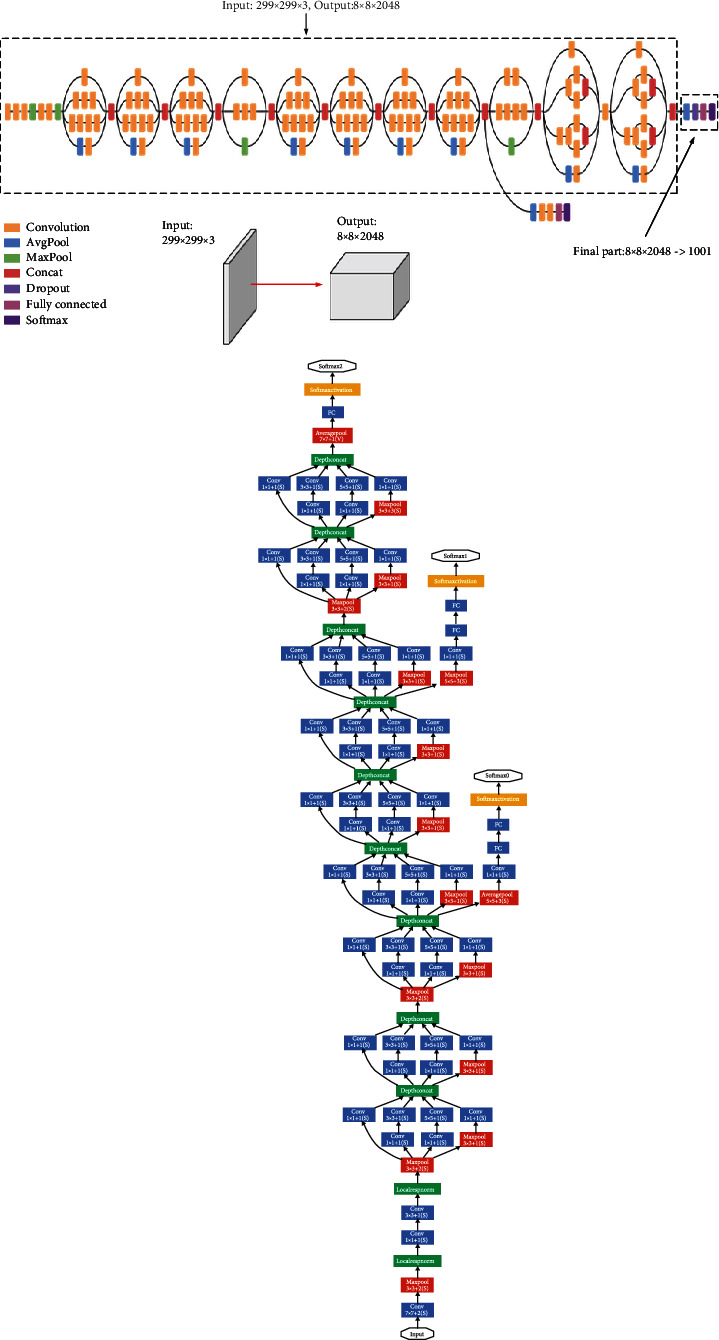
Google's TensorFlow inception neural network.

The Tensorflow model is a neural network with several hidden layers. Each layer performs different transformations on an input image. At the last hidden layer (bottleneck), the network summarizes enough information for the output layer, which performs the classification task. The model uses Google's ImageNet, which was trained to distinguish between objects of 1000 different classes [35, 36]. In this paper, the feature extraction capabilities for the Tensorflow classifier using the inception model, as depicted in Figure 7, were reused. Google's TensorFlow inception neural network architecture depicted in Figure 7 was found to be very useful for transfer learning capabilities. The last top layer was removed, and a new classification layer was trained on top using the food images dataset. The food dataset used contained 25 classes, each with 300 images. The same number of images was used to prevent overfitting. 80% of the dataset was used for training, 10% for validation, and the final 10% for testing. The classifier was trained with 1200 training steps initially and then with 4000 steps to improve accuracy. 10 images were randomly selected from the training set for each step and fed to the bottleneck layer to obtain a prediction. The predictions obtained from each step were compared with the actual labels to update the weights using the backpropagation algorithm. An estimate of how well the classifier will perform in a classification task was obtained using the testing dataset.

### 3.5. The Activity and Reminder Module

**Figure 8:**
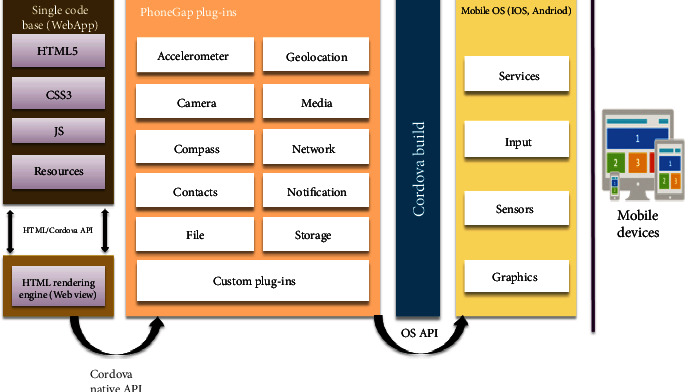
Architecture for Cordova plugin.

This module uses Cordova plugins to communicate with the operating system and the rendered WebView in Figure 8.

#### 3.5.1. Activity Tracker

This module encourages users to exercise using Google maps and geolocation. The distance travelled by the user is recorded and saved to the firebase database. The user selects the destination, which is converted to longitude and latitude coordinates using geocoder. The route is drawn for the user, and based on the speed of the user, the time used for the activity is determined.

#### 3.5.2. Medication Reminder

Many times, humans forget to perform certain obligations; forgetting to take diabetes medications or glucose readings will have serious consequences. This module provides a reminder platform that uses Ionic notifications to notify users when it is time to take their medications. Users add their medications using the Ionic calendar, and notifications are sent to them for the period selected by the user. An interface is also provided for users to log their blood glucose readings. A pdf of the readings can be generated, and logged data can also be visualized using high charts. The system architecture for the Cordova plugin upon which the Ionic notifications and other services are based is shown in Figure 8, and it incorporates the web technologies, sensors, and graphics for the mobile OS. It provides an HTML5 rendering engine with Phone Gap plugins for mobile app development.

## 4. System Implementation and Testing

To save and update the user's information in real-time, Google's firebase was set up and implemented. A firebase project was created in the Firebase console, a setup user authentication and storage rules were generated using a configuration file for both Android application and web application. A JSON file containing meals and the nutrition content was then imported. To extend the reach of the application, there was the need to design a cross-platform application not limited to a particular Operating System. To achieve this, the Ionic framework, which uses AngularJS built on top of Cordova, was used to implement the system. Ionic was used because it has mobile-friendly controls that make cross-platform apps feel like native apps and has a lot of documentation. The navigation logic to enable users to move from one view to the other was implemented and firebase and Google services configuration file added to allow communication with the database. Finally, the various Cordova plugins needed were installed, and the interfaces implemented using HTML, JavaScript, and other Ionic components. To recognize food images, images of different local dishes were collected, labelled, and put into classes. Tensorflow for CPU support was installed, and the Inception model trained with the dataset. To test the classifier, a Python script was written to set up a Flask server that will provide an interface for retrieving classification results. Two graphs were generated after training the model, one containing the retrained model and the other containing the output labels. The trained graphs were imported into a Python function that is called to obtain predictions. Another function was implemented in the Flask server for the meal recommendation. This function loads user's information and meals from the firebase database, performs K-NN to find the best meals for the user, and sends the top best five meals to the database, which syncs data to the front end in real-time. For the question and answer bot, a Microsoft QnA service was created with a knowledge base which extracts question and answers pairs from pdfs with information of diabetes and URLs of diabetes forums.

After successfully implementing the various modules, the system was integrated. This was achieved in 2 steps, namely,

1. Making HTTP requests from the front end to the Microsoft model and Flask web service
2. Adding the Firebase configuration file to the front end and the Flask app

### 4.1. Testing and Results

Based on the design and development of the component modules of the diabetes management system, the testing results are categorized into (1) Diabetes Intelligent Meal Recommender Module results, (2) Educational Module for Diabetics with Food Recognition Engine results, (3) Activity Tracking Module results, and (4) Medication Reminder Module results.

**Figure 9:**
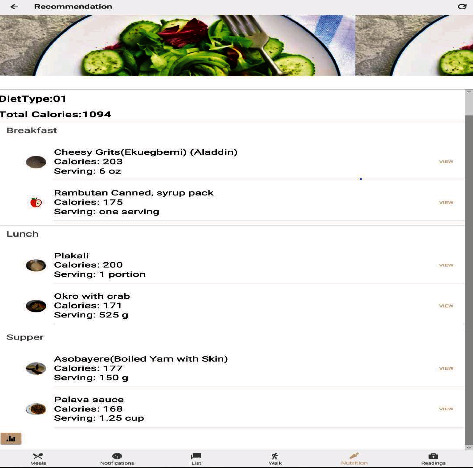
Meal recommendation with the calorific content and amount of serving.

The Diabetes Intelligent Meal Recommender module recommended five (5) excellent local and continental dishes for users based on their calorific needs and the amount of serving to meet their calorie requirements, as shown in Figure 9. The dataset used to train the model was collected from two sources, namely, data from social media and smartphone cameras. The images obtained using this approach were very few and mostly of low quality. To obtain high-quality images for training and validation of our model, a web search and crawler script for different food categories, written in Python, was used. The Python beautiful soup package was used to download images for each label. The downloaded images were filtered to remove ambiguous and duplicate images to reduce cross-category noise. A total of 25 classes was obtained, with each category containing 300 images each. The same number of images was used for each category to prevent overfitting. The dataset was partitioned as follows: 80% of the collected image dataset was used for training, and 10% was used for validation and 10% for testing of the model.

**Figure 10:**
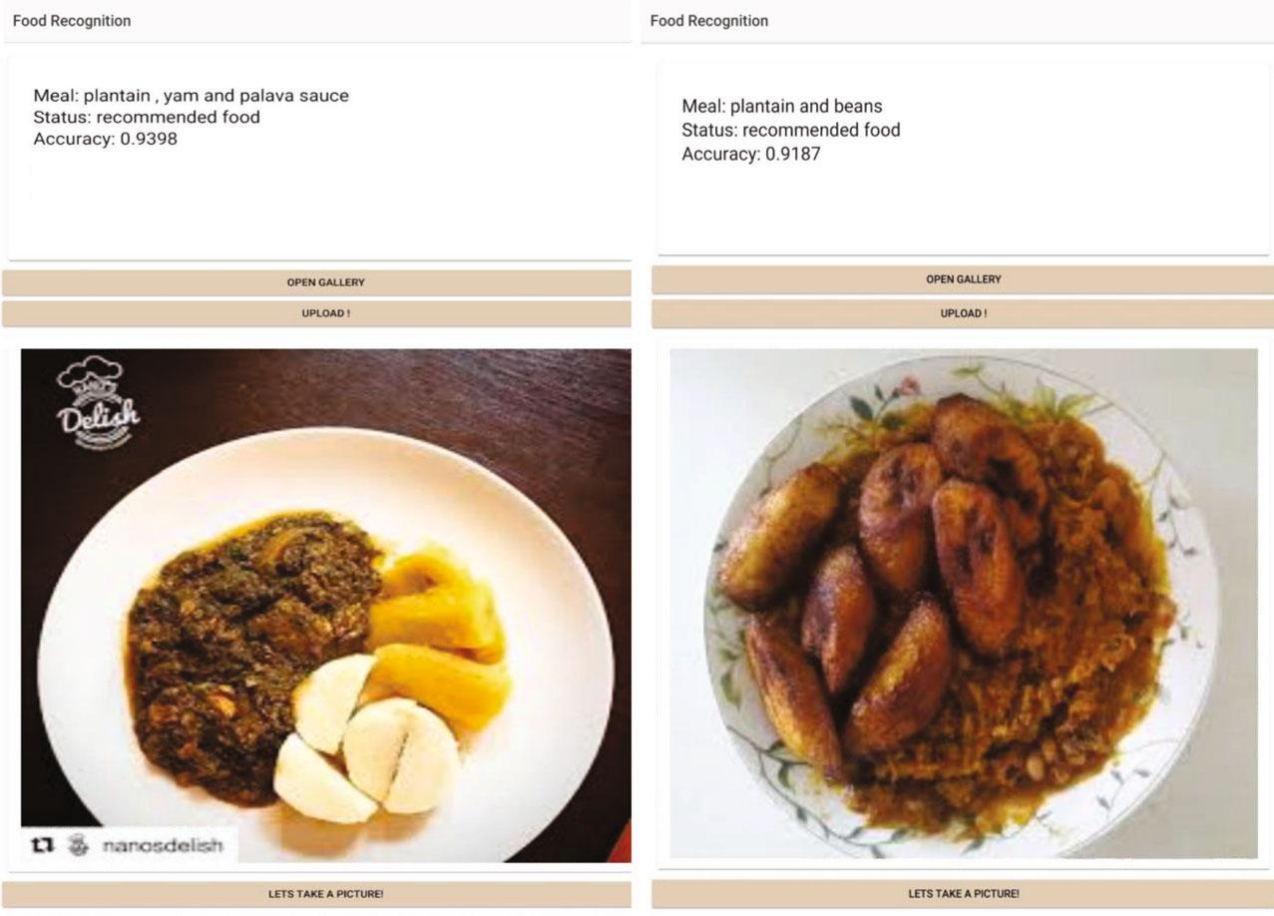
Testing the food recognition model with local dishes.

**Figure 11:**
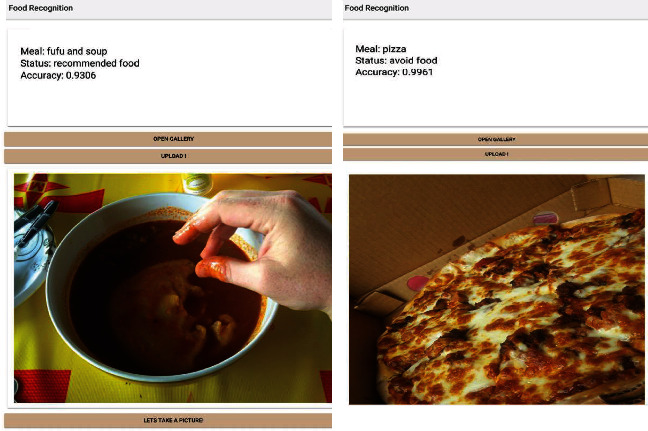
Testing the food recognition model for local (fufu and soup) and continental dishes.

**Figure 12:**
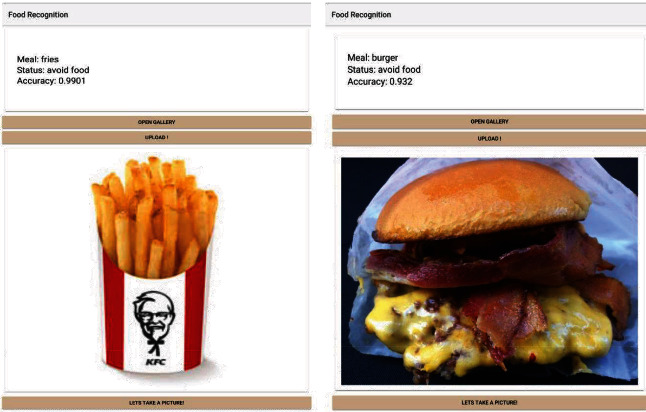
Testing the food recognition model for fries and cheeseburger.

The prediction and accuracy obtained for the food recognition model when tested with a high-quality image and low-quality images are shown in Figures 10, 11, and 12 below. Figure 9 shows a meal generated for an individual with an 1100 calorie requirement. The model generates a meal with 1094 calories with a ratio of carbs within the 40-65 percentile, and fats and protein within the 10% to 30% as recommended by the World Health Organization.  Figure 10 shown below obtained a 93.98% accuracy level for plantain and palava sauce and 91.87% for plantain and beans, which are part of the recommended diet for diabetes to control blood sugar. The quality of the images used in training enhanced prediction accuracy. The model can perform efficiently, but it is subject to how well it is trained. Inadequate representation of the categories results in low performance in accuracy levels.  Figure 11 shows a 93.06% accuracy level for fufu and palm nut and 99.61% accuracy level for pizza recognitions while clearly delineating the recommended food for diabetes. For a diabetic patient, pizza is not recommended due to the fact it is highly processed food, and the body lacks the right insulin amount for digestion and absorption, thereby triggering high blood sugar levels. The high accuracy level is attributable to the fact that these images are of high quality, and they were also used in training the food recognition classifier model and presented a good representation of the category.

**Figure 13:**
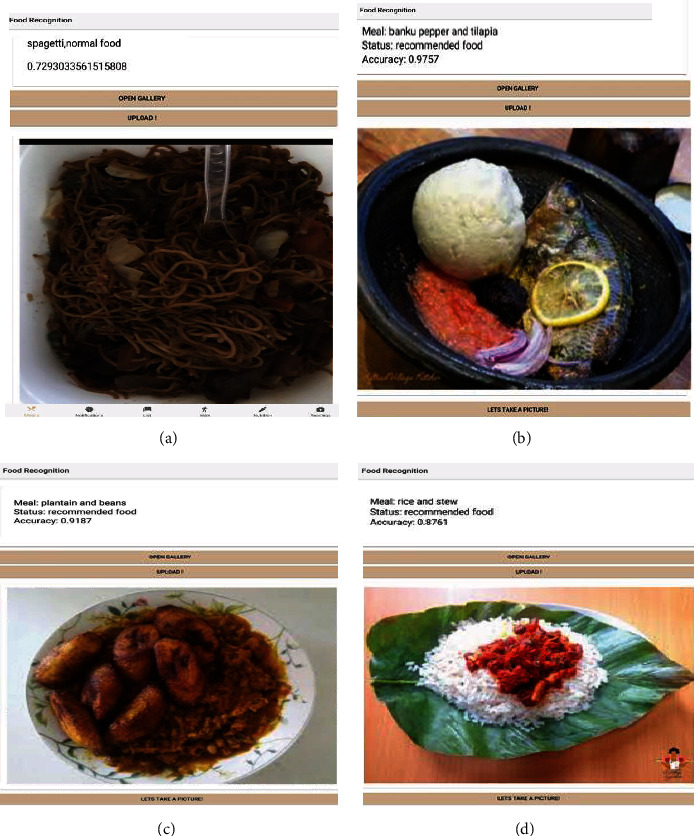
Testing the food recognition model.

Figure 12 above was not used during training, but it was 99% and 93.2% accurate during the food recognition module results. This is because fries and cheeseburger are not similar to other classes; images have high-quality pixels and a good representation of the images that were used in the training phase of the development.  Figure 13(a) had a lower accuracy level because low-quality images were not used in training. This made the classifier perform with only a 72.9% accuracy level for the low-quality image. In the case of Figures 13(b)–13(d), the accuracy levels increased due to the quality of the images used in training and validation as well as testing. The food recognition model gave accurate results for recommended local and international dishes for people with diabetes.

**Figure 14:**
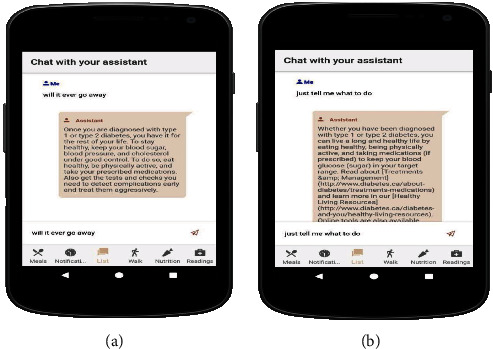
Testing question and answer bot.

Figure 14, shown in the appendix, shows the assistant answers the question correctly and in a human-like nature. Because a lot of questions and answers were used in the training and validation of the model, the model returns no answer found only in instances where the question is not related to diabetes.

**Figure 15:**
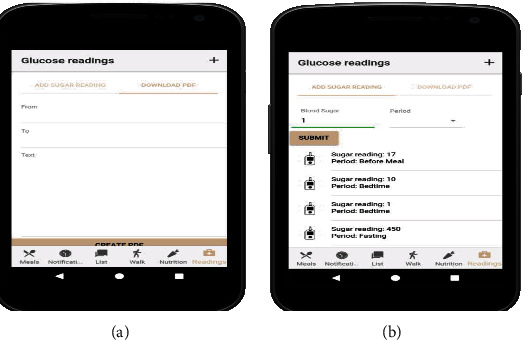
Glucose readings on mobile phone for diabetes management.

**Figure 16:**
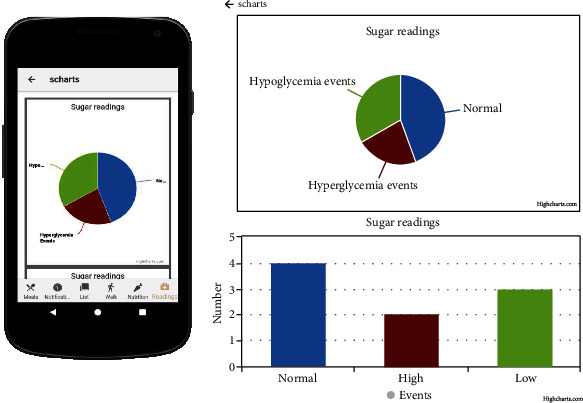
Charts of Glucose reading to track progress on a daily, monthly, or yearly level.

**Figure 17:**
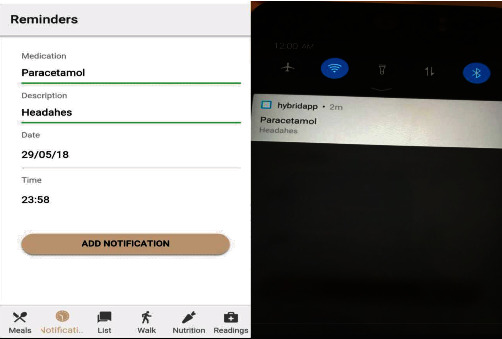
Notification and Reminders for taking medications.

Figure 15 in the appendix enables blood glucose readings and tracking on a mobile phone for effective diabetes management. It logs all the blood glucose data for analytics and charts your activity and monitors your progress, as shown in Figure 16 in the appendix. Notification and tracking of reminders for taking medication are shown in Figure 17 in the appendix.

### 4.2. Performance Evaluation

**Figure 18:**
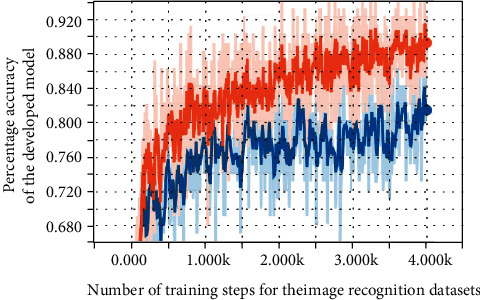
Accuracy of the model of the inception model.

The accuracy obtained for different training steps after training the Inception model on the parameters is shown in Figure 18 below for the different steps used in training.

The error for the various training steps was also determined using a cross-entropy loss function. The accuracy improved with an increasing number of steps, but the cross-entropy reduces as the number of steps increases. Although increasing the number of steps increases accuracy and reduces the error, the accuracy does not improve after a certain point. Most Tensorflow models perform better when trained with distortions by performing various transformations on the images. This model did not perform well with distortions because increasing the contrast of images like fried rice can change the colour to orange and cause it to be misclassified as Jollof rice.

## 5. Conclusion and Recommendation

This research paper has presented a meal recommendation system with food recognition capabilities which focused on generating daily personalized meal plans for the users, according to their nutritional necessities and previous meal preferences. The reviewed literature presented some gaps which informed the design and development of an integrated diabetes management platform for patients using K-Nearest Neighbour (KNN) algorithm, a supervised machine learning model for food recommendation system for diabetics, (2) scheduling and reminding diabetic patients to take their medication and blood glucose readings for doctor's intervention via mobile app, (3) encouraging and tracking the activity of diabetic patients, and (4) providing an interactive visual interface to help them make meaning of their readings and establishing a sufficient connection between the doctor and the diabetic patient using e-mail and chatbots. These integrated technologies present state-of-the-art solutions for the effective management of diabetes. This research paper required us to provide a framework with a user-friendly interface for people with diabetes to monitor their diet, medication, and activity levels. The task has been solved using state of the art algorithms in artificial intelligence.

The proposed framework factors the diabetes management problem into subgoals: building a Tensorflow neural network model for food classification; thus, it allows users to upload an image to determine if a meal is recommended for consumption; implementing K-Nearest Neighbour (KNN) algorithm to recommend meals; using cognitive sciences to build a diabetes question and answer chatbot; tracking user activity, user geolocation and generating pdfs of logged blood sugar readings. The food recognition model was evaluated with cross-entropy metrics that support validation using neural networks with a backpropagation algorithm. The model learned features of the images fed from local Ghanaian dishes with specific nutritional value and essence in managing diabetics and provided accurate image classification with given labels and corresponding accuracy. The model achieved specified goals by predicting with high accuracy, labels of unseen new images. The food recognition and classification model achieved over 95% accuracy levels for specific calorie intakes. The performance of the meal recommender model and question and answer chatbot was tested with a designed cross-platform user-friendly interface using Cordova and Ionic Frameworks for software development for both mobile and web applications. The system recommended meals to meet the calorific needs of users successfully using KNN (with *k* = 5) and answered questions asked in a human-like way.

The implemented system would solve the problem of managing activity, dieting recommendations, and medication notification for diabetics. The critical limitation of this work is that it does not address corresponding hardware modules for insulin pumps and control, as discussed by others in the review, and that may constitute a fatal limitation since insulin control is crucial. It concentrates principally on developing software for diabetes management with a machine learning algorithm. Other supervised and unsupervised machine learning algorithms, such as Support Vector Machines, random forests, K-Means, and Fuzzy C-Means, could be explored as well.

Finally, there is hope that this system will be useful to people with diabetes now and in the future. The focus of this work has been on implementing a software system that will take into consideration the various factors that affect diabetics. The most crucial issue was to get different models to work; consequently, there are improvements to make. The nonfatal limitations include a lack of wearables for physical activity tracking and associated model to determine the number of calories burned from each activity undertaken. The present system tracks the user's walk and saves the route but does not relate the saved route to the calories burned. In the future, the calories burned would be determined, and the various modules will work together to predict the user's future blood glucose readings.

## Data Availability

Data available on request.
